# Reliability and validity study of the Spanish adaptation of the “Wijma Delivery Expectancy/Experience Questionnaire” (W-DEQ-A)

**DOI:** 10.1371/journal.pone.0248595

**Published:** 2021-03-19

**Authors:** Celia Maria Ortega-Cejas, Juan Roldán-Merino, Teresa Lluch-Canut, Mª Isabel Castrillo-Pérez, Mª Mercedes Vicente-Hernández, Marta Jimenez-Barragan, Ainoa Biurrun-Garrido, Mariona Farres-Tarafa, Irma Casas, Sandra Cabrera-Jaime

**Affiliations:** 1 Midwife, Sexual and Reproductive Health Clinic (ASSIR), Mollet del Vallès, Barcelona, Spain; 2 Campus Docent, Sant Joan de Déu - Fundació Privada, School of Nursing, University of Barcelona, Barcelona, Spain; 3 Research Group GIES (Grupo de investigación en Enfermería, Educación y Sociedad), Barcelona, Spain; 4 Research Group GEIMAC (Consolidated Group 2017-1681: Group of Studies of Invarianza of the Instruments of Measurement and Analysis of Change in the Social and Health Areas), Barcelona, Spain; 5 Faculty of Medicine and Health Sciences, University of Barcelona, Barcelona, Spain; 6 Midwife, Sexual and Reproductive Health Clinic (ASSIR) La Riera, Badalona, Barcelona, Spain; 7 Midwife, Sexual and Reproductive Health Clinic (ASSIR) Doctor Barraquer, Sant Adrià del Besòs, Barcelona, Spain; 8 Midwifery Coordinator, Sexual and Reproductive Health Clinic (ASSIR) Fundació Assistencial Mútua Terrassa, (Terrassa) Midwife, Sexual and Reproductive Health Clinic (ASSIR) Rambla Terrassa, (Terrassa) Docente en Fundació Universitaria del Bages, Barcelona, Spain; 9 Member Research Group GRISIMula (Grupo emergente 2017 SGR 531; Grupo en Recerca Enfermera en Simulación), Barcelona, Spain; 10 Secretary, GRISCA Research Group (Nurising Simulation in Catalonia and Andorra Research Group), Barcelona, Spain; 11 Universitat Autònoma de Barcelona, Barcelona, Spain; 12 Preventive Medicine Service, Hospital Germans Trias i Pujol, Barcelona, Spain; 13 Research Group Innovation in Respiratory Infections and Tuberculosis Diagnosis (Group Consolidat 2017 SGR 494), Barcelona, Spain; 14 Corporate Care Management, Institut Català d’Oncologia (ICO), L’Hospitalet de LLobregat, Barcelona, Spain; 15 GRIN Group, IDIBELL, Institute of Biomedical Research, Barcelona, Spain; Murcia University, SPAIN

## Abstract

The Wijma Delivery Expectancy/Experience Questionnaire (W-DEQ-A) is an instrument that evaluates fear of childbirth through the expectations of women in relation to childbirth and their experience during the birth. The objective of this study was to translate the W-DEQ-A into Spanish and analyse its reliability and validity. The study was carried out in two phases: (1) adapting the questionnaire to Spanish and (2) a transversal study in a sample of 273 pregnant women in the Sexual and Reproductive Health centres in the Metropolitan Northern Barcelona in Catalonia (Spain). The psychometric properties were analysed in terms of reliability and construct validity. The confirmatory factorial analysis did not confirm the unidimensionality of the original structure of the WDEQ-A, as happened with the other studies in which it has previously been validated. The result of the exploratory factorial analysis suggests four factors, or dimensions, very similar but not identical to those obtained in other analysis studies of the W-DEQ-A. The Cronbach alpha and the omega scale were also adequate for all the scales and for each of the dimensions. The results of this study confirm the findings of other studies that suggest that the W-DEQ-A is multi-dimensional. In the Spanish version of the W-DEQ-A four dimensions have been identified to explore fear of childbirth in pregnant women. The Spanish version of the WDEQ-A (WDEQ-A-Sp) is reliable and valid for the measurement of fear of childbirth in clinical practice and for use in future research.

## Introduction

Pregnancy and future childbirth is one of the most important life events in the life of a woman and the experience of birth can be defined as a complex individual life process that incorporates psychological and profound subjective physiological processes [1]. Most women experience feelings of anxiety or worry about the development and wellbeing of the pregnancy, the baby and about the birth throughout the pregnancy [2]. However, this worry focussed on the birth can set off feelings of anxiety or intense fear [3, 4]. In some pregnant women this emotion can lead them to have feelings of avoidance of the birth that coincide with the definition of phobia according to the diagnostic criteria of the DSM-V [5–7]. The term *tokophobia* is used to refer to pathological fear of childbirth [8] and is defined as that which affects or interferes with the everyday life of pregnant women [9, 10]. Tokophobia is classified as primary if it affects nulliparous women, or secondary if it affects multiparous pregnant women [5]. However, consensus does not exist on the definition of fear of childbirth [11], or on the measuring tool to detect it [12], so in the literature it is described as light, moderate or severe [13].

The prevalence of fear of childbirth has been difficult to ascertain due to the wide variability of results in different studies carried out in different countries and populations, as well as a consequence of varying definitions referenced on fear, describing rates varying from 3.7% to 43% for prevalence [14]. For this reason, a systematic review carried out by O’Connell estimated global fear of childbirth at 14% [14]. Prevalence of fear of childbirth is also different in function of the births a woman has experienced and greater in nulliparous women than in multiparous women [15].

While stress, anxiety, depression and a lack of social support have been related to high levels of fear in nulliparous women, having a negative experience at a previous birth is what causes the greatest fear of childbirth amongst multiparous pregnant women [16], as is the case of those who have been subjected to a previous caesarean or vacuum-assisted births [17]. Equally, other factors have been associated with a greater level of fear of childbirth such as: a history of mental illness [18], history of sexual abuse [19] and low self-esteem [20].

Similarly, several studies have analysed the elements that make up fear of childbirth. Women refer to their worries being related to fear of the unknown, the possibility that they or the baby suffer injuries, fear of pain, fear of loss of control, doubts about their ability to give birth or a lack of support from health providers [21, 22].

Fear of childbirth has been associated with a higher risk of elective caesarean [17, 23–25], higher risk of emergency caesarean [26, 27] and increased risk of suffering post-traumatic stress during the post-natal period [28, 29]. It has also been associated with greater use of epidural anaesthetic [30, 31], prolonged labour [32] and greater probability of dystocia during pushing [26].

During recent years various instruments have been developed to screen for fear of childbirth. Nevertheless, the questionnaire most used for fear of childbirth is the Wijma Delivery Expectancy/Experience Questionnaire (W-DEQ) and it is the only one that evaluates antepartum and postpartum fear [12].

The W-DEQ questionnaire was published by Wijma et al. in 1998. It is a self-administrated questionnaire; it has 2 versions with 33 items each, which evaluate fear of childbirth through the expectations of women in relation to the birth (WDEQ A) and the experience of stress after the birth (WDEQ B).

Since the questionnaire was developed it has been translated into several languages and has been used in different studies to explore fear of childbirth [23, 33–47]. However, although it was conceived as a unidimensional instrument, different analyses carried out in the different validations have shown a multifactorial structure [13, 33, 34, 37, 39, 41–47].

Table 1 shows the languages and populations where it has been validated and the principal characteristics of each validation.

**Table 1:** Summary of the characteristics of the WDEQ validated in the different languages and countries.

Author	Language; Country (year)	Sample	Type of analysis	Number of factors (items)	Factors labels	Item	Reliability
Johnson & Slade [34]	English; UK (2002)	443	EFA	4 (31)	Fear	6,19,17,12,5,2,16,8,22,24,20,25,4,27,10	.91
					Isolation	15,7,3,11	
					Lack of positive anticipation	14,18,13,21,1,29,30,23	
					Riskiness	33,32,31,9	
Wiklund et al. [37]	Swedish; Sweden	496	EFA	4 (33)	Fear	6,4,5,17,29,22,30,8,12,9,26,10,19,16,2,7,31	No data
					Lack of positive anticipation	18,14,13,21,1,28,23,15,3	
					Isolation	25,24,27,20,11	
					Riskiness	32,33	
Fenwick et al. [13]	English; Australia (2009)	401	EFA	4 (32)	Fear	17,19,12,2,6,16,5,4,24,25,10,28	.91
					Isolation	14,13,18,30,22,21,29,23,1,9	
					Lack of positive anticipation	15,7,11,3,20,8,31,27	
					Riskiness	32,33	
Garthus-Niegel et al. [39]	Norway (2011)	1680	CFA	6 (25)	Fear	6,12,19,20,24,27	.75-.87
					Negative appraisal	1,13,14,18	
					Loneliness	3,7,15	
					Lack of self-efficacy	4,5,9,10,16,22,26	
					Lack of positive anticipation	28,29,30	
					Concerns for the child	32,33	
Takegata et al., [41]	Japanese (2013)	231	EFA	4 (33)	Fear	19,25,16,27,12,17,22,2,6,4,5,24,10,	.70
						28,26,23,29,30	
					Isolation	7,11,3,15,8,20	
					Lack of positive anticipation	18,13,14,9,21,1	
					Riskiness	32,33,31	
Fenaroli et al., [33]	Italian (2013)		EFA	4 (16)	Fear	6,19,2,24,12,25,27,8	No data
					Negative feelings	13,18,14	
					Lack of confidence	22,23,9	
					Negative thoughts	32,33	
Fenaroli et al., [33]	Italian (2013)	500	CFA	3 (14)	Fear,	6,19,2,24,25,27,8,12	.86
					Negative feelings	13,18,14	
					Lack of confidence	22,23,9	
Lukasse et al. [47]	Norwegian	6870	EFA	6 (33)	Lack of self-efficacy	5,22,10,17,4,16,9,23,12,26	.92
	Swedish						
	Danish						
	Estonian				Loneliness	7,11,15,20,8,3,6	
	Flemish				Negative appraisal	14,18,13,21,1	
	Icelandic						
	Russian						
	(2014)				Lack of positive anticipation	29,30,28,31	
					Fear	25,27,24,19,2	
					Concerns for the child	32,33	
Pallant et al. [48]	Australia (2016)	1410	CFA	4 (17)	Negative emotions	2,6,8,12,19	.82-.87
			EFA				
			Rash Analysis				
					Lack of positive emotions	5,9,17,18,23	
					Social Isolation	3,7,11,15	
					Moment of birth	28,29,30	
Mortazavi, F. [42]	Farsi; Iran (2017)	405	EFA	6 (32)	Lack of self-efficacy	13,10,9,5,14,17,18,23,22,4	.91
					Lack of positive anticipation	28,21,29,30	
					Loneliness	15,11,8,7,3,2,20,31	
					Fear	19,6,24,16,12	
					Concerns for the child	32,33	
					Concerns about losing control	27,25,26	
Abedi et al. [46]	Persian; Iran (2017)	200	EFA	9 (33)	Despair	3,7,8,11,12,15,20	.64
					Confidence	4,5,9,13,16,22	
					Fear	2,6,19,24	
					Happiness	14,18,21,23,28	
					Loss of control	25,27,31	
					Independence	1,10,17	
					Concern about new-born	32,33	
					Obvious	29,30	
					Control	26	
MoghaddamHossein et al. [45]	Hungarian; Hungary (2018)	343	EFA	4 (30)	Isolation	11,15,3,20,31,27,25,8,19,2	.92
					Lack of positive emotions	17,5,13,22,16,4,10,9,1,23,14	
					Moment of birth	28,24,30,21,18	
					Fear	6,12,7,24	
Andaroon et al. [43]	Persian; Iran (2020)	220	EFA	6 (31)	Lack of self-efficacy	4,5,10,13,14,16,17,20,22,23	.84
					Fear	3,6,7,8,11,12,15	
					Negative appraisal	18,21,26,28	
					Lack of positive anticipation	2,19,25,27	
					Concerns about child	30,32,33	
					Loneliness	1,9,29	
Khwepeya et al. [44]	Malawi (2020)	264	EFA	3 (26)	Not reported	Not reported	No data
	Malawi (2020)	264	CFA	3 (23)	Fear	7,15,12,11,6,3,8,2,20,25	.84
					Negative appraisal	14,17,31,1,18,13,9,16	
					Lack of self-efficacy	21,22,26,23,4,28,5,10	
Pitel et al. [49]	Slovak; Slovakia (2020)	279	EFA	7 (33)	Lack of composure	16,17,12,26,10,24,25	.93
					Negative appraisal	13,18,14,21,23,9	
					Lack of self-efficacy	5,4,22,27,21,8	
					Lack of positive anticipation	30,29,28	
					Fear and hopelessness	20,19,31,11,6	
					Loneliness	*3*,*7*,*15*	
					Concern for the child	32,33	

Although fear of childbirth is a socially recognized phenomenon in Spain, it is not clinically studied during the development of the pregnancy because there is no validated questionnaire available in Spanish [50]. Given the prevalence indicated in other countries and the perinatal repercussions it brings; a validated tool to carry out screening for fear of childbirth for pregnant women in Spain is needed. This would allow midwives to detect it and carry out interventions to reduce the repercussions for the benefit of the health of the pregnant woman and the newborn.

To achieve this, the objective of this study was to translate into Spanish and analyse the reliability and validity of the Wijma Delivery Expectancy/Experience Questionnaire (W-DEQ-A).

## Methods

### Design

The study was carried out in two phases. In the first phase, the W-DEQ-A questionnaire was adapted to Spanish: in the second phase the metrics of the version translated to Spanish were analysed.

### Participants and setting

The sample for the study was made up of 273 pregnant women in the Sexual and Reproductive Health Clinics in the Northern Metropolitan region in Barcelona in Catalonia (Spain). The questionnaires were administered to pregnant women during routine prenatal visits in the 34 weeks of gestation. To complete the questionnaires, pregnant women were asked to answer how they thought they would feel during labour and how they imagined labour would be. Pregnant women over 18 years of age and who did not present language difficulties in reading and completing the questionnaire in Spanish were selected. Women with a history of perinatal death were excluded.

The women were recruited consecutively during the study period between January 2019 and January 2020.

The size of the sample was calculated from the recommendations of various authors who recommended between 5 and 20 participants for each item featuring on the questionnaire [51, 52]. In this study it was agreed to include 10 participants for each item featuring on the questionnaire. As a consequence of the COVID-19 pandemic, the study finished in January 2020 as it was considered that fear of COVID-19 at the time surrounding childbirth among the pregnant women from this date onwards could be a factor that influenced the results. Finally, 8 pregnant women were included for each item on the questionnaire. The sample of 273 participants was deemed adequate to carry out the study.

### Variables and source of information

All the items on the W-DEQ-A questionnaire were included as variables. It is a questionnaire made up of 33 items, which in the original version are grouped in one single dimension.

Each item is evaluated using an ordinal scale of 0 to 5. The extremes of the replies (0 and 5 respectively) correspond to the opposites of a feeling or thought. The minimal score is 0 and the maximum is 165. Scores over 85 indicate severe fear of childbirth and scores over 100 indicate clinical signs of fear of childbirth. In the original Wijma study [53] a Cronbach alpha of 0.87 was obtained.

Other variables were also collected such as: age, level of education, employment status, number of births and presence or not of a partner.

### Procedure

The cultural adaptation process of version A of questionnaire W-DEQ was carried out according to the Standards for Educational and Psychological Testing [54]. Prior to starting the translation the author of the questionnaire’s permission was sought for its adaption for the Spanish population.

The English version of the questionnaire provided by the author was translated to Spanish by independent Spanish sworn translators, whose mother-tongue was Spanish and who were fully competent in English, providing two versions of the questionnaire in Spanish W-DEQ-A, which were evaluated by a committee of experts made up of a gynaecologist, a psychologist specialised in the area of sexual and reproductive health, 3 midwives and a specialist research nurse. This version was sent to two new sworn translators unfamiliar with the original version whose mother tongue was English and fully competent in Spanish for the retro-translation to English. The two versions obtained were compared with the original questionnaire by the same committee of experts, who found no discrepancies that required modifications. Table 2 shows the semantic equivalence of items from English to Spanish.

**Table 2:** Shows the semantic equivalence of items from English to Spanish that were metrically validated on the W-DEQ-A-Sp.

Item	English	Spanish
Item 1	Fantastic	Fantástico
Item 2	Frightful	Horrible
Item 3	Lonely	Sola
Item 4	Strong	Fuerte
Item 5	Confident	Confiada
Item 6	Afraid	Asustada
Item 7	Deserted	Desatendida
Item 8	Weak	Débil
Item 9	Safe	Segura
Item 10	Independent	Independiente
Item 11	Desolate	Desolada
Item 12	Tense	Tensa
Item 13	Glad	Contenta
Item 14	Proud	Orgullosa
Item 15	Abandoned	Abandonada
Item 16	Composed	Íntegra
Item 17	Relaxed	Relajada
Item 18	Happy	Feliz
Item 19	Panic	Pánico
Item 20	Hopelessness	Desesperanza
Item 21	Longing for the child	Deseosa del bebé
Item 22	Self-confidence	Autoconfianza
Item 23	Trust	Confianza
Item 24	Pain	Dolor
Item 25	I will behave extremely badly	Me comportaré estremadamente mal
Item 26	I allow my body to take total control	Permitiré a mi cuerpo tomar el control total
Item 27	I will totally lose control of myself	Voy a perder el control total de mi misma
Item 28	Enjoyable	Agradable
Item 29	Natural	Natural
Item 30	Should be	Como debe ser
Item 31	Dangerous	Peligroso
Item 32	Fantasies that your child die during labour/delivery	Fantasías sobre si el bebé se muere durante el parto
Item 33	Fantasies that your child will be injured during labour/delivery	Fantasías de que su bebé sufrirá lesiones durante el parto

### Pretest

A pretest was carried out with a total of 30 pregnant women with the aim of evaluating the clarity and understanding of the items and the format and time for completion. The pregnant women concluded that it was easy to understand and required little time, between 10 and 15 minutes to complete it. After the debriefing it was not necessary to make any changes in either the format or the content. The Spanish version was named W-DEQ-A-Sp.

### Statistical analysis

First a confirmatory factorial analysis (CFA) was carried out to test the unidimensional model of the original scale proposed by Wijma [53] in 1998 and then an exploratory factorial analysis was performed to determine the number of factors in the Spanish version following the same procedure as has been used to adapt the questionnaire to the different languages for which it has been validated [55]. The following adjustment indices were calculated to determine the general adjustment of the model: the chi-squared goodness-of-fit test, the ratio between chi-squared and the degrees of freedom (χ2/df), the Comparative Fit Index (CFI), the Goodness-of-Fit Index (GFI), the Adjusted Goodness-of-Fit Index (AGFI) and the Root Mean Square Error of Approximation (RMSEA). The criteria for a good fit were CFI, GFI and AGFI values above 0.90 [56–58], and RMSEA values were to be below 0.08 [55, 59].

Before using the EFA its suitability was tested using the Kaiser Mayer Olkin test (KMO) and the Bartlett sphericity test. For the extraction of the factors three basic rules were kept in mind (a) Kaiser rule [60] retaining the components with values greater than 1; (b) the graphic inspection of scree plot [61], in which all components above the curve are removed/excluded and (c) the classical implementation of Horn’s Parallel Analysis [62], a method that adequately identifies the number of components of the questionnaire [63].

The EFA was adjusted to the polychoric correlation matrix given the ordinal nature of the items [64]. The communalities and coefficients in the matrix were also checked and coefficients greater than 30 were considered significant.

The adjustment function chosen for the data extraction method was weighted least squares with correctional adjustment statistics for mean and variance [65]. The factors were rotated using the Robust Promin rotation [66].

The reliability was analysed using the internal consistence evaluated with the Cronbach alpha and omega Index. Values were considered appropriate with a Cronbach’s alpha value greater than 0.70 [67]. Values that oscillate between 0.70 and 0.90 are considered adequate [68, 69], values greater than 0.90 are considered excellent [70]. Values were considered appropriate with an omega Index scale value greater than 0.80 [71]. Temporary stability or test-retest was evaluated after 2 weeks from the intra-class correlation coefficient in a sample of 257 pregnant women. The values of this coefficient oscillate between 0 and 1. The concordance is considered to be excellent when the coefficient is greater than 0.90, good if it is between 0.71 and 0.90, mediocre between 0.31 and 0.50 and poor when it is less than 0.31 [72–74].

CFA models were estimated using structural equation modelling (EQS 6.4 for Windows, Multivariate Software, Inc., Encino, CA, USA) and EFA was carried out using the Factor Analysis programme [75].

### Ethical considerations

The study was approved by the Human Research Ethics Committee at the Germans Trias i Pujol Hospital (code PI14-074) and by the Medical Research Ethics Committee Jordi Gol (code P14/106). All the participants were informed of the aims of the study and gave their verbal and written consent and they participated voluntarily. The translation was completed with the express consent of the original author of the questionnaire.

## Results

### Characteristics of participants

The characteristics of the participants is shown in Table 3. A total of 273 pregnant women were included in the study. The average age was 33.0 (SD 5.0) with a range of 20 to 46 years. The 65.2% were nulliparous and 3.3% declared that they had no partner. 78.0% referred to university studies and 88.6% to stable work.

**Table 3:** Participant characteristics.

Characteristics	n	%
Parity		
Nulliparous	178	65,2
Multiparous	95	34,8
Partner		
With partner	264	96,7
Without partner	9	3,3
Level of studies		
Primary	19	7,0
Secondary	41	15,0
University	213	78,0
Employment status		
Working	242	88,6
Out of work	31	11,4

### Construct validity

Here we present the different analyses carried out to evaluate the construct validity, confirmatory factorial analysis (CFA) and exploratory factorial analysis (EFA).

### Confirmatory factor analysis (CFA)

Confirmatory factorial analysis (CFA) was used to verify the unidimensional structure of the original version of the questionnaire. In Table 4 the single factor model adjustment is shown, which contains 33 items from the questionnaire WDEQ-A-Sp. The model showed a deficient adjustment (for example CFI = 0.59 and RMSEA of 0.10). These results did not confirm the unidimensionality of the original structure of the questionnaire of WDEQ-A.

**Table 4:** Goodness-of-fit indices for the confirmatory model W-DEQ-A-Sp.

INDEX	VALUE
CFI	0.594
GFI	0.918
AGFI	0.907
RMSEA	0.109
Goodness of fit test	χ^2^ = 2102,020; *gl* = 495; *P* < 0.0001
Reason for fit	χ^2^ / *gl* = 4,24

CFI: Comparative Fit Index. GFI: Goodness of Fit Index. AGFI: Adjusted Goodness of Fit Index. RMSEA: Root Mean Standard Error of Approximation.

### Exploratory factor analysis (EFA)

**Fig 1:**
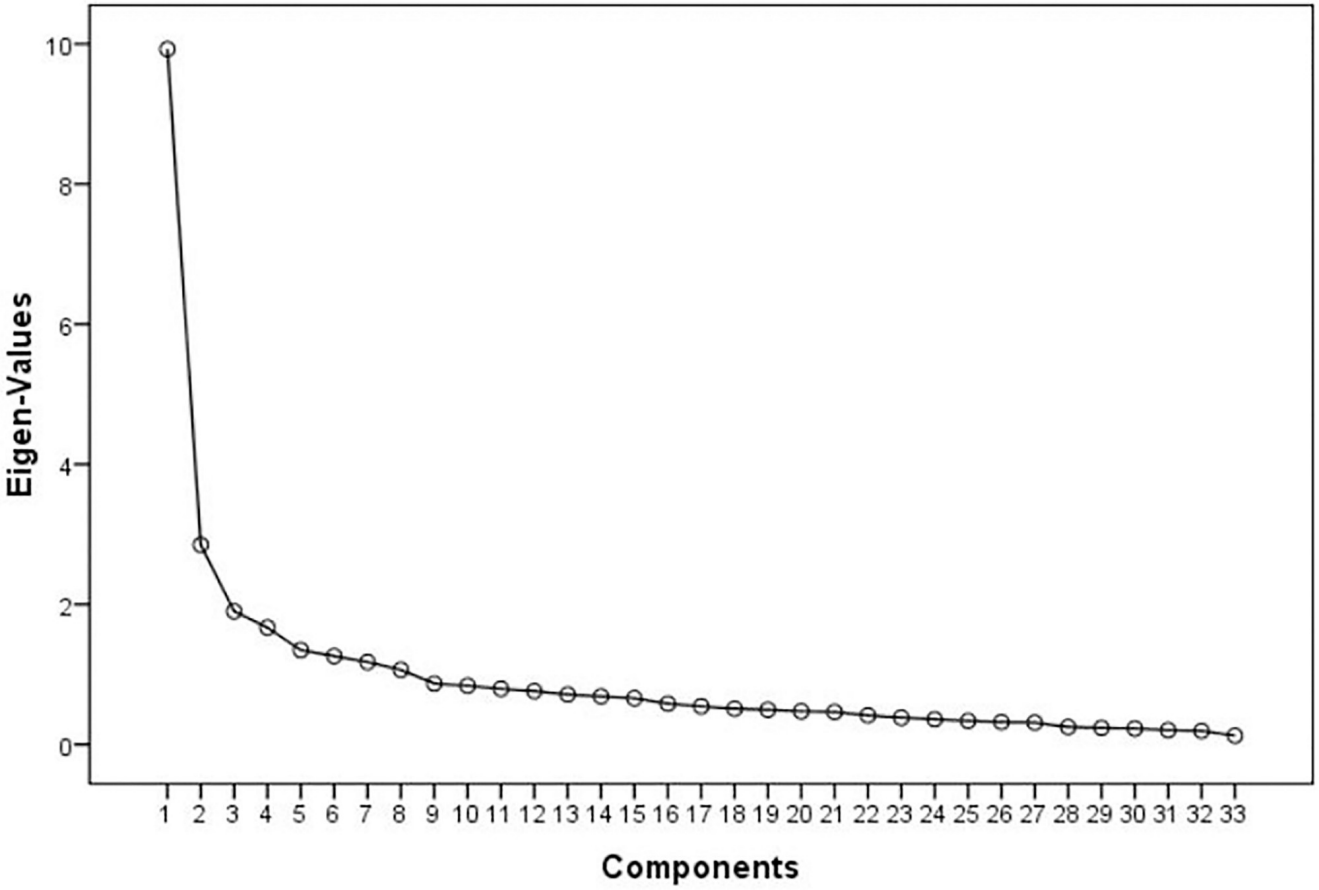
Scree plot of the W-DEQ-A-Sp.

Exploratory factor analysis (EFA) was carried out of the Spanish sample to test if subcategories of fear exist within the W-DEQ-A-Sp as has been done for the other different languages for which the questionnaire has been validated. The previous analysis identified 2 items with a value less than 0.30 (item 26 and item 27) that were eliminated. Seven factors had auto-values greater than 1, which explains the 69.0% of variance. However, the scree plot (Fig 1) and the results of the parallel analysis suggested 4 values for which the real data autovalues exceeded the random data autovalues. 55.3% of the variance is explained by these 4 factors.

Table 5 shows the goodness of fit indexes for the 4 factor model, which are excellent.

**Table 5:** Indices of goodness of fit of the exploratory factor analysis to the model for four dimensions the W-DEQ-A-Sp.

INDEX	VALUE	95% confidence interval
CFI	0.993	0.993–0.996
GFI	0.982	0.980–0.987
AGFI	0.976	0.974–0.983
RMSEA	0.034	0.010–0.050
Goodness of fit test	χ^2^ = 454,600; *gl* = 347; *P* < 0.0001	
Reason for fit	χ^2^ / *gl* = 1,31	

**CFI**: Comparative Fit Index. **GFI**: Goodness of Fit Index. **AGFI**: Adjusted Goodness of Fit Index. **RMSEA**: Root Mean Standard Error of Approximation.

The 4 factors defined as “fear”, “isolation”, lack of positive anticipation” and “riskiness” in the UK study [34] were similarly defined in the Spanish sample. Table 6 shows the percentage of variance explained for each factor and the variables that configure each one. To facilitate the interpretation they have been ordered in function of size and factorial loading.

**Table 6:** Loading matrix related to the exploratory factor analysis solution.

Item No.	Description	Factor 1	Factor 2	Factor 3	Factor 4
		‘fear’	‘isolation’	‘lack of positive anticipation’	‘riskiness’
19	Panic	0.873			
6	Afraid	0.799			
17	Relaxed	0.743			
9	Safe	0.712			
12	Tense	0.661			
5	Confident	0.635			
24	Pain	0.600			
10	Independent	0.569			
4	Strong	0.561			
2	Frightful	0.554			
8	Weak	0.530			
1	Fantastic	0.501			
15	Abandoned		0.841		
11	Desolate		0.645		
3	Lonely		0.599		
7	Deserted		0.568		
20	Hopelessness		0.398		
25	I will behave extremely badly	.	0.326		
13	Glad			0.735	
14	Proud			0.700	
18	Happy			0.698	
21	Longing for the child			0.674	
30	Should be			0.632	
28	Enjoyable			0.592	
22	Self-confidence			0.574	
29	Natural			0.568	
23	Trust			0.463	
16	Composed			0.335	
33	Fantasies that your child will be injured during labour/delivery				0.894
32	Fantasies that your child die during labour/delivery				0.851
31	Dangerous				0.341
Percent of variance		**35,34**	**9,77**	**6,51**	**5.60**

### Internal consistency and temporal stability

The Cronbach’s alpha for the total of the questionnaire was 0.91 and values greater than 0.70 were obtained in all the factors making up the questionnaire. The omega coefficient (ω) for the total questionnaire and for each of the factors was greater than 0.81.

ICC analysis demonstrated that the test–retest reliability was 0.91 (95% confidence interval 0.89–0.93) and this value was greater than 0.84 for the four dimensions.

In Table 7 the results of the W-DEQ-A-Sp are shown related to reliability and the test-retest temporal stability.

**Table 7:** W-DEQ-A-Sp Cronbach’s alpha coefficient, omega coefficient and ICC test-retest (n = 257).

Factor	Cronbach’s alpha	Omega (ω)	ICC (CI 95%)
**F.1**. Fear	0.885	0.900	0.903 (0.876–0.924)
**F.2**. Isolation	0.732	0.830	0.855 (0.815–0.887)
**F.3**. Lack of positive anticipation	0.868	0.894	0.861 (0.822–0.891)
**F.4**. Riskiness	0.719	0.819	0.849 (0.807–0.882)
Total	0.918	0.936	0.917 (0.894–0.935)

**ICC**: intraclass correlation coefficient; **CI**: Confidence interval.

## Discussion

The objective of this study was to translate the Wijma Delivery Expectancy/Experience Questionnaire (W-DEQ-A) into Spanish and analyse the reliability and validity of the Spanish version. The original questionnaire designed by Wijma contains 33 items grouped in one single dimension. It was developed to “measure fear of childbirth by means of the woman’s cognitive appraisal regarding the delivery” [53] (p.85).

In our study we initially carried out a CFA using the generalized least squares method with the aim of determining if the scores reproduced the unidimensional structure on which the original questionnaire is based. Regarding the adjustment indexes of the model: Comparative Fit Index (CFI), Goodness of Fit Index (GFI), Adjusted Goodness of Fit Index (AGFI), Root Mean Square Error of Approximation (RMSEA), and normalized Chi-squared all presented a deficient adjustment, therefore we concluded that the model does not fit conveniently. These results are consistent with many other studies on the lack of unidimensionality of the WDEQ-A [13, 33, 34, 48].

Because of this, we had to abandon the hypothesis of a single factor and explore our sample to determine which model should be expected in the Spanish population. For this we also carried out an EFA. We used the classical implementation of Horn’s Parallel Analysis [62]. This method is superior to the conventional methods for correctly identifying the true number of dimensions [62, 63, 76]. The results of the analysis have suggested 4 factors, or dimensions, which are similar to, but not identical to those obtained in other factor analysis studies of WDEQ-A [13, 33, 34, 38, 41, 48].

However, of all the studies which have identified four factors, that which was most similar to ours was that of the United Kingdom (UK) [34]. In both studies 31 items have presented a factorial load superior to 0.30 and have grouped together in four dimensions in a very similar way.

The explained variance of the structure with four dimensions was 55%. This variance was very similar to that found in the majority of studies that have validated this questionnaire [13, 33, 34, 37, 41–44, 47] and was only less than that found in the study carried out by Abedi et all., Moghaddam Hosseim et al. y Pitel et al. [45, 46, 49]. Although the percentage of explained variance found in this study could be considered to be low, it is not currently recommended to use the interpretation of explained variance as the only indicator of factors identified. Rather it is recommended to incorporate procedures based on Parallel Analysis, which selects common components or factors that present own values higher than those expected by chance, as for example, the Minimum Average Partial test, or the RMSEA adjustment indicator [77, 78]. In this study both Parallel Analysis and the RMSEA have been used to identify the adequate number of factors.

To analyse the reliability of the questionnaire an analysis of its internal consistency was carried out using the Cronbach’s alpha coefficient. The total Cronbach’s alpha value for the questionnaire was 0.91 (considered excellent), with factors varying between 0.71 and 0.88, so that all the dimensions were adequate.

The fact that the internal consistency is greater than 0.90 can be interpreted as meaning that there are redundant elements in the questionnaire. However, according to Kottner et al. (2011), for an instrument to be able to be used to take clinical decisions, the minimum Cronbach’s alpha acceptable should be 0.90 [79]. Furthermore, these values are very similar to those obtained in other studies in which the reliability was measured with the Cronbach’s alpha coefficient [13, 34, 42, 45, 47, 49]. Additionally in this study the homogeneity coefficient was calculated for the corrected items estimating the correlations of each item with the total scale and with its corresponding subscale. In this study a correlation of 0.20 was accepted as the inferior limit [80].

In this study the reliability has also been analysed using the omega coefficient (ω) which is recommended when one dimension has few items. Factor four (Riskiness) consists of three items, so it was decided to calculate this coefficient as a complementary method. Values greater than 0.80 are considered adequate. The omega coefficient in this study was satisfactory for both the total questionnaire and each of the four dimensions with values greater than 0.80 for all of them [71].

The temporal stability or test-retest has also been analysed in this study. The temporal stability of the WDEQ-A questionnaire has not been checked in any other validation study. Of the 273 pregnant women who participated in this study 257 completed the questionnaire again on a separate occasion after two weeks. The ICC obtained for the total questionnaire and for each of the dimensions was good with values greater than 0.80 for each of the dimensions of the questionnaire [72–74].

### Limitations

This study has several limitations that should be taken into account. Firstly, all the pregnant women who participated in the study did so voluntarily and were selected consecutively by their midwives, so the selection could be biased. However, a large number of pregnant women from different centres in Barcelona were included and their sociodemographic and clinical characteristics are very similar to the rest of the pregnant women in the Spanish population so that these results can be generalized. Secondly, sensitivity to change has not been studied in the Spanish population, but it would be interesting to research this in future longitudinal or post-intervention studies.

## Conclusions

The Spanish version of the WDEQ-A (WDEQ-A-Sp) is the first instrument to be validated in Spanish for the screening of fear of childbirth. The results of this study confirm the findings of other studies that suggest that the WDEQ-A is multi-dimensional. In the Spanish version of the WDEQ-A-Sp four dimensions have been identified that make it possible to explore fear of childbirth in pregnant women (fear’, ‘isolation’, ‘lack of positive anticipation’ and ‘riskiness’). It is a multidimensional questionnaire, which is easy to complete and with good psychometric properties in terms of reliability and construct validity; making it suitable for implementation in clinical practice. More studies with a larger sample size and developed in other areas of Spain are needed to assess the prevalence of fear of childbirth in the Spanish population. Likewise, having a validated instrument would allow future research to be carried out into fear of childbirth in order to implement interventions to reduce it. Finally, the statistical techniques used in this study allow us to add together solid evidence to back up the use of this questionnaire in the Spanish population.

## Supporting information

S1 Data (XLSX)
